# Tau PET following acute TBI: Off-target binding to blood products, tauopathy, or both?

**DOI:** 10.3389/fnimg.2022.958558

**Published:** 2022-10-14

**Authors:** Tracy Butler, Gloria C. Chiang, Sumit Narayan Niogi, Xiuyuan Hugh Wang, Carly Skudin, Emily Tanzi, Nimmi Wickramasuriya, Jonathan Spiegel, Thomas Maloney, Silky Pahlajani, Liangdong Zhou, Simon Morim, Henry Rusinek, Marc Normandin, Jonathan P. Dyke, Edward K. Fung, Yi Li, Lidia Glodzik, Qolamreza Ray Razlighi, Sudhin A. Shah, Mony de Leon

**Affiliations:** 1 Department of Radiology, Weill Cornell Medicine, New York, NY, United States; 2 New York University Grossman School of Medicine, New York, NY, United States; 3 Department of Radiology Harvard Medical School, Boston, MA, United States

**Keywords:** traumatic brain injury (TBI), acute TBI, complicated mild TBI, Positron Emission Tomography (PET), Chronic Traumatic Encephalopathy (CTE), neurodegeneration, tau, neurofibrillary tau

## Abstract

Repeated mild Traumatic Brain Injury (TBI) is a risk factor for Chronic Traumatic Encephalopathy (CTE), characterized pathologically by neurofibrillary tau deposition in the depths of brain sulci and surrounding blood vessels. The mechanism by which TBI leads to CTE remains unknown but has been posited to relate to axonal shear injury leading to release and possibly deposition of tau at the time of injury. As part of an IRB-approved study designed to learn how processes occurring acutely after TBI may predict later proteinopathy and neurodegeneration, we performed tau PET using 18F-MK6240 and MRI within 14 days of complicated mild TBI in three subjects. PET radiotracer accumulation was apparent in regions of traumatic hemorrhage in all subjects, with prominent intraparenchymal PET signal in one young subject with a history of repeated sports-related concussions. These results are consistent with off-target tracer binding to blood products as well as possible on-target binding to chronically and/or acutely-deposited neurofibrillary tau. Both explanations are highly relevant to applying tau PET to understanding TBI and CTE. Additional study is needed to assess the potential utility of tau PET in understanding how processes occurring acutely after TBI, such as release and deposition of tau and blood from damaged axons and blood vessels, may relate to development CTE years later.

## Introduction

Chronic Traumatic Encephalopathy (CTE), a neurodegenerative disorder associated with progressive neuropsychiatric decline in individuals who have suffered repeated mild traumatic brain injury (TBI), is currently diagnosable only at autopsy based on the presence of neurofibrillary tau tangles in the depths of brain sulci and surrounding blood vessels (McKee et al., 2014). Positron Emission Tomography (PET) now allows assessment of tauopathy *in vivo*. A small number of tau PET studies have revealed tau deposition months to years following TBI (repeated mild and single moderate-severe) and in subjects with clinically-probable CTE, (Stern et al., 2019; Ayubcha et al., 2021; Marklund et al., 2021) though differences between patients and controls are subtle, confounded by non-specific binding of early generation tracers, and not considered diagnostic at the individual level. The mechanism by which repeated TBI leads to CTE remains unknown but has been posited to relate to release of tau at the time of injury through axonal shearing. Tau levels in blood, CSF and interstitial fluid rise rapidly following TBI and can provide clinically relevant information about TBI severity and prognosis (Zetterberg, 2017). We designed a study to learn if tau PET performed shortly after TBI can improve understanding of how processes occurring acutely after TBI may predict later neurodegeneration. We report early results from this study, focusing on the first subject enrolled whose tau PET scan was markedly abnormal with prominent radiotracer uptake in regions of hemorrhage.

## Methods

### Participants and setting

As part of a study approved by the Weill Cornell Medicine IRB, adult subjects with complicated mild [GCS 13–15 with trauma-related CT abnormality such as intracranial hemorrhage or contusion (Marshall et al., 1991)] or moderate TBI were recruited from the Weill Cornell—New York Presbyterian Emergency Department to undergo PET and MRI neuroimaging within 14 days of injury. All subjects provided informed consent prior to participation.

### Neuroimage acquisition

PET scans were acquired from 0 to 60 and 90 to 120 min on a Siemens Biograph PET/CT after rapid IV bolus injection of ~555 MBq ^18^F-MK6240.

MRI was acquired on a research-dedicated 3T GE system. Sequences included T1 BRAVO for PET coregistration (TR of 256*8.2 = 2,097 ms, TE = 3.2 ms. TI = 450 ms, Acquired Resolution = 1 × 1 × 1 mm interpolated to 0.5 × 0.5 × 0.5 mm; 180 sagittal slices) and 3D Spiral Arterial Spin Labeling (ASL; TR = 4,851 ms, TE = 10.6 ms, Acquired Resolution = 1.875 × 1.875 × 3.8 mm, 36 axial slices).

### Neuroimage processing and analysis

After reconstruction and decay correction, PET images were motion corrected and coregistered with T1 MRI using validated methods (Tahmi et al., 2019). SUVr images for summed 90–120 min acquisition were generated with reference to cerebellar gray matter, defined by Freesurfer and eroded by 2 voxels to avoid partial volume effects.

## Results

**FIGURE 1:**
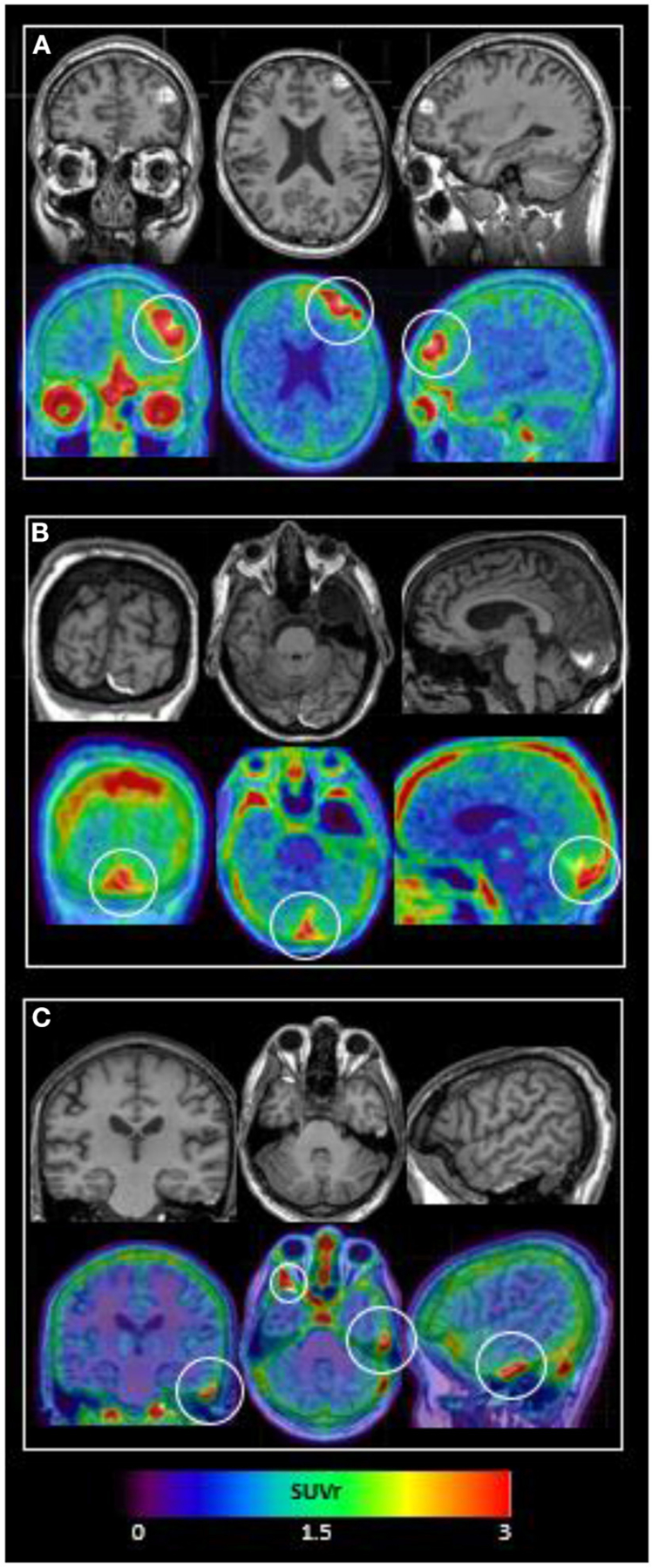
^18^F-MK6240 PET and MRI performed within 14 days of complicated mild TBI in 3 subjects. Top panel shows T1 MRI and bottom panel shows ^18^F-MK6240 PET (SUVr) overlaid on MRI. **(A) Subject #1:** MRI shows hemorrhagic contusions in L frontal and L>R orbitofrontal regions. PET shows focal radiotracer accumulation within and surrounding these regions, with the L frontal lesion (white circle) showing a c-shaped configuration. **(B) Subject #2:** MRI shows parafalcine trace subdural hematoma, diffuse atrophy, and a developmental arachnoid cyst. PET signal in the region of subdural hematoma (white circle) may be present, but is difficult to differentiate from (normal, off-target) signal in adjacent dura. **(C) Subject #3:** MRI shows R anterior and L lateral temporal epidural hematomas. PET shows extra-axial radiotracer accumulation in these regions (L temporal = small white circle; R temporal = larger white circle).

TBI subject characteristics are shown in Table 1. All subjects suffered complicated mild TBI with brief (<5 min) loss or alteration of consciousness with normal Glasgow Comas Scale (GCS) scores of 15 by the time of Emergency Department evaluation. As shown in Figure 1, all subjects’ ^18^F-MK6240 PET scans were abnormal. Subject #1, with a history of repeated (10+) sports-related concussions, had two frontal areas of intense ^18^F-MK6240 uptake in the regions of MR-visible T1 hyperintense, hemorrhagic cerebral contusion and subdural hemorrhage. In this subject only, radiotracer accumulation appeared to extend into brain parenchyma. Subject #2 had probable extra-axial tracer accumulation along a parafalcine subdural hematoma adjacent to (normal off-target) tracer accumulation in skull. Subject #3 showed a small amount of extra-axial tracer accumulation in the region of epidural hematomas.

## Discussion

^18^F-MK6240 tau PET performed shortly after complicated mild TBI in three subjects revealed focal radiotracer accumulation in regions of MR-visible hemorrhagic injury. This has not previously been reported. Prominent increased PET signal in one subject with a history of multiple prior concussions suggests the possibility that these results correspond to acute and/or chronic tauopathy. This would be relevant to understanding the pathogenesis of CTE, which to date has proven difficult to image using PET, and which remains impossible to diagnose *in vivo*. However, alternate explanations for ^18^F-MK6240 accumulation in regions of traumatic hemorrhage such as tracer extravasation through a damaged blood-brain barrier (BBB) and off-target radiotracer binding must be considered.

### Tracer extravasation

^18^F-MK6240 is highly BBB permeable [a general requirement for brain PET radiotracers (Pike, 2009)] and does not require BBB breakdown to enter brain parenchyma. Arterial spin labeling (ASL) MRI showed decreased blood flow to regions of radiotracer accumulation (data not shown) which would be expected to decrease tracer delivery. Other PET studies of acute TBI show *decreased* radiotracer accumulation in regions of hemorrhagic injury (Langfitt et al., 1986). We therefore do not think focal radiotracer accumulation can be attributed to tracer leakage or extravasation.

### Binding to blood products

While ^18^F-MK6240 is considered specific for neurofibrillary tau, autoradiography has shown weak binding to blood products (Aguero et al., 2019; Malarte et al., 2021). Consistent with this, all three subjects showed radiotracer accumulation outside the brain, in regions of extra-axial hemorrhage. This cannot represent parenchymal tau deposition, and confirms that off-target binding to blood products must be considered when interpreting ^18^F-MK6240 PET. Additional studies e.g., autoradiography and immunohistochemical studies in human tissue obtained at surgery or postmortem and from animal models are needed to clarify the tissue target of ^18^F-MK6240 in the setting of hemorrhage.

### Tauopathy

In Subject #1, PET signal extends into brain parenchyma and well beyond regions of MRI-visible hemorrhage, and we question whether tracer binding to blood products can account fully for these findings. We propose that tau PET signal in this subject with a history of multiple prior concussion, scanned shortly after complicated mild TBI, corresponds, at least in part, to on-target binding of ^18^F-MK6240 to acutely and/or chronically deposited neurofibrillary tau. While tau tangles are not generally considered to form quickly after TBI, this has been demonstrated in teenaged athletes who died days after suffering concussions, with tau localized to regions of microhemorrhage (McKee et al., 2014; Tagge et al., 2018). This finding in concussion, along with the perivascular location of chronically-deposited tau in CTE (McKee et al., 2014) support the theory that traumatic microvascular injury and release of blood components into brain parenchyma is important to the pathophysiology of tauopathy after TBI (Michalicova et al., 2017; Sandsmark et al., 2019). Much additional neuroimaging and other work is needed to determine if our PET finding of tau radiotracer accumulation surrounding hemorrhagic contusion also supports this theory (vs. corresponding solely to off-target binding) and understand relevant factors, e.g. whether this finding was detectable in one subject because of a predisposing history of multiple prior concussions, injury features, genetic and/or other factors, and whether PET abnormalities resolve over time. The absence of similar tau PET findings in subjects scanned later after injury (Ayubcha et al., 2021; Marklund et al., 2021) suggest this apparent lesion may be transient; follow-up scans are planned for 1 year after injury.

## Conclusion

^18^F-MK6240 tau PET imaging performed shortly after complicated mild TBI in three subjects revealed areas of focal tracer accumulation in regions of traumatic hemorrhage consistent with 1) off-target radiotracer binding to blood products and/or 2) post-traumatic tau deposition. Both explanations are relevant to advancing understanding of tauopathy in TBI and CTE. It is notable that evidence of possible parenchymal tauopathy was present only in one subject with a history of multiple prior concussion. Additional study is needed to clarify the tissue target of ^18^F-MK6240 PET in the setting of TBI complicated by intracranial bleeding, and to assess its potential utility in understanding how processes occurring acutely after TBI, such as release and deposition of tau and blood from damaged axons and vessels, may relate to future neurodegeneration.

## Figures and Tables

**TABLE 1:** Subject characteristics.

	Age/sex	Injury	Initial head CT	TBI history
1	Male, 30’s	Fall (sports)	Left frontal hemorrhagic contusions; small volume subarachnoid and subdural hemorrhage	>10 prior sports concussions
2	Female, 70’s	Tripped on sidewalk	Right orbital floor fracture; small subdural hematoma along the posterior falx and left tentorium	No prior TBI
3	Male, 30’s	Fell off bike; no helmet	Skull fracture; small bilateral temporal epidural and small right frontal intraparenchymal hemorrhages	No prior TBI

## Data Availability

The raw data supporting the conclusions of this article will be made available by the authors, without undue reservation.
